# Bone Regeneration and Integration Following Implantation of Octacalcium Phosphate/Gelatin Composite After Benign Bone Tumor Surgery

**DOI:** 10.7759/cureus.98541

**Published:** 2025-12-05

**Authors:** Hirotaka Kurata, Yu Mori, Shinichirou Yoshida, Jun Iwatsu, Katsuhiro Fukushima, Ryo Hamai, Osamu Suzuki, Toshimi Aizawa

**Affiliations:** 1 Orthopedic Surgery, Tohoku University Graduate School of Medicine, Sendai, JPN; 2 Orthopedic Surgery, Tohoku university Graduate School of Medicine, Sendai, JPN; 3 Division of Biomaterials Science and Engineering (Division of Craniofacial Function Engineering), Tohoku University Graduate School of Dentistry, Sendai, JPN

**Keywords:** benign bone tumor, bone graft substitute, bone regeneration, curettage, octacalcium phosphate/gelatin composite

## Abstract

Bone defect reconstruction following benign bone tumor curettage remains challenging due to limitations of traditional grafts. Autologous bone provides excellent osteoconductivity and osteoinductivity, but it causes donor site morbidity. In contrast, allogeneic and xenogeneic grafts are limited by infection risk and restricted availability. Synthetic substitutes such as hydroxyapatite and β-tricalcium phosphate offer osteoconductivity but lack osteoinductive potential. Octacalcium phosphate (OCP), a precursor of biological apatite, possesses both osteoconductivity and intrinsic osteoinductivity and exhibits greater resorbability than conventional materials. The OCP/gelatin composite (OCP/Gel) was recently approved for clinical use in Japan.

This prospective study investigated seven patients who underwent OCP/Gel implantation after curettage of benign bone tumors. No internal fixation was required, and no complications related to the material occurred. Radiographic follow-up demonstrated a gradual decrease in radiolucency at the graft site, and computed tomography at six months confirmed trabecular bone formation in all cases.

These findings suggest that OCP/Gel is progressively replaced by new bone and can promote early bone regeneration after tumor curettage. Despite the small sample size, single-center design, and limited defect size, OCP/Gel appears to be a safe and effective bone substitute. Further multicenter studies are warranted to confirm its long-term efficacy and optimal clinical indications.

## Introduction

Bone defects resulting from fractures, infections, or tumor resection represent a major clinical challenge in orthopedic surgery. Although autologous and allogeneic bone grafts remain the gold standard for reconstruction, they are limited by donor site morbidity, restricted availability, and the potential risk of donor-derived infections [1,2]. Synthetic bone graft substitutes have therefore attracted attention as alternative graft materials, offering a stable supply and eliminating donor-associated complications. Conventional substitute materials, such as hydroxyapatite (HA) and β-tricalcium phosphate (β-TCP), exhibit osteoconductivity but lack osteoinductivity, thereby limiting their capacity to induce bone regeneration in large or poorly vascularized defects [2-6].

Octacalcium phosphate (OCP), regarded as a precursor of biological apatite [7], exhibits both high osteoconductivity and intrinsic osteoinductivity [8]. Furthermore, OCP demonstrates greater resorbability than HA and β-TCP [3,7,9-13,14], facilitating dynamic remodeling and replacement by new bone. The development of mass production technology for an OCP/gelatin composite (OCP/Gel) has enabled its practical use. The OCP/Gel product (Bricta, Nipro, Osaka, Japan) was officially approved for insurance coverage and clinical application in Japan in October 2024. A porous OCP/gel composite material has been developed, and its bone-healing ability has been investigated in rat critical-size calvarial defects [15], standardized long-bone defects in rabbits [16,17], and ovariectomized rats [18]. The OCP/gel composite possesses a porous structure, and even when the gelatin component is thermally cross-linked, the crystalline structure of OCP is preserved. This characteristic structure has been confirmed by X-ray diffraction analysis. In preclinical studies, OCP/Gel has shown remarkable osteogenic performance in various challenging bone defect models, including rat and rabbit cortical through-defects and miniature swine spinal fusion models [16,17,19]. These studies demonstrated that OCP/Gel promoted robust new bone formation and favorable integration compared with conventional calcium phosphate materials.

There have been reports on the effectiveness of HA/Col in treating iliac crest defects following spinal fusion surgery [20]. Nevertheless, currently available biomaterials such as HA/collagen composites (HA/Col) remain insufficient for complete bone regeneration in large segmental defects [21], highlighting the need for more biologically active materials capable of inducing bone remodeling comparable to autologous bone. Against this background, OCP-based composites have emerged as promising candidates for next-generation bone substitutes. The purpose of the present study was to elucidate the bone regenerative capacity of OCP/Gel in bone defects following benign bone tumor resection in clinical settings.

## Materials and methods

Bone graft substitute

The OCP/Gel used in this study is characterized by its high bone-regenerative potential derived from OCP and its excellent handling properties resulting from the elasticity provided by gelatin.

Patients

This prospective observational study investigated seven consecutive patients who underwent bone graft substitute implantation using OCP/Gel following curettage for primary benign bone tumors at Tohoku University Hospital between October 2024 and March 2025. In the hemangioma case, no bone wax or preoperative embolization was used. Hemostasis was achieved with electrocautery before packing the OCP/Gel into the cavity. All surgeries were completed early enough within the recruitment period to allow six-month postoperative CT follow-up for all patients prior to data analysis.

The study was conducted in accordance with the ethical standards of the Declaration of Helsinki and was approved by the Ethics Committee of Tohoku University Hospital (approval number 2024-1-659). Written informed consent was obtained from all participants prior to enrollment.

Clinical data

Clinical data, including patient age, sex, tumor location, histological diagnosis, tumor size, amount of implanted OCP/Gel, preoperative treatment history, and the presence or absence of pathological fracture, were collected prospectively from medical records. Tumor size was evaluated using both the maximum diameter and calculated volume based on preoperative computed tomography (CT) images. All radiographic measurements were performed using a digital measurement tool in the Picture Archiving and Communication System (Vue PACS, version 12.2.5.4000286; Carestream Health, Rochester, NY, USA).

Radiographic and CT evaluation

Plain radiographs of the operated sites were obtained immediately after surgery, and at one week, one month, two months, four months, and six months postoperatively. Bone regeneration was assessed based on the gradual disappearance of radiolucency and the continuity of trabecular bone at the graft site. CT was performed at six months postoperatively to confirm trabecular bone formation and cortical continuity. All images were independently reviewed by two orthopedic surgeons, and discrepancies were resolved by consensus.

Data and statistical analysis

Given the small sample size and the absence of a control group, the analysis was descriptive in nature. Continuous variables were summarized as medians and ranges, and categorical variables were presented as counts and percentages. No inferential statistical tests were performed. Missing data were minimal and handled by complete-case analysis.

This study was designed and reported in accordance with the STROBE (Strengthening the Reporting of Observational Studies in Epidemiology) guidelines. Because this was a single-arm observational case series without a control group, statistical comparisons, confounding factor adjustments, and sensitivity analyses were not applicable.

## Results

The cohort consisted of five males and two females (n = 7), with a mean age of 15.3 years. Three patients were diagnosed following pathological fractures, all of which had achieved bone union through conservative treatment prior to surgery. Anhydrous ethanol treatment was performed in three cases. One patient had a recurrent giant cell tumor of bone, whereas the remaining six cases involved primary bone tumors. No additional internal fixation using metallic implants was performed in any of the cases.

Curettage of the bone lesion was performed in all cases, followed by filling of the defect with the OCP/Gel. In selected cases, additional anhydrous ethanol treatment was applied to the cavity wall after curettage, depending on the tumor type and intraoperative findings. The graft material was manually packed into the defect and molded to fit the cavity without the need for metallic fixation. Clinical information of the patients is summarized in Table 1. A total of seven patients (five males and two females; mean age, 15.3 years; range, 8-38 years) were included in this study (Table 1).

**Table 1:** Summary of cases Age is expressed in years. “Positive” and “Negative” indicate the presence or absence of each condition (ethanol treatment, previous treatment, or pathological fracture).

	Age	Sex	Diagnosis	Location	Diameter (mm)	Volume (mL)	OCP/Gel (mL)	Ethanol treatment	Previous treatment	Pathological fracture
1	38	F	Fibrous dysplasia	Middle phalanx of the index finger; metaphysis	11.8	0.4	0.6	Negative	Negative	Negative
2	12	M	Giant cell tumor of bone	Second metatarsal; metaphysis	16.4	1.9	2	Positive	Positive	Negative
3	17	M	Desmoplastic fibroma of bone	Middle phalanx of the middle finger; metaphysis	18.7	1.2	0.75	Positive	Negative	Negative
4	13	F	Non-ossifying fibroma	Fibula; metaphysis	31	4.1	6	Negative	Negative	Negative
5	8	M	Simple bone cyst	Humerus; metaphysis	31.7	11.8	8	Positive	Negative	Positive
6	9	M	Hemangioma of bone	Tibia; diaphysis	34.5	9.4	6	Negative	Negative	Positive
7	10	M	Non-ossifying fibroma	Humerus; diaphysis	64	40.7	16	Negative	Negative	Positive

The diagnoses consisted of fibrous dysplasia (n = 1), giant cell tumor of bone (n = 1), desmoplastic fibroma of the bone (n = 1), non-ossifying fibroma (n = 2), simple bone cyst (n = 1), and hemangioma of the bone (n = 1). Lesions were located in the phalanges of the hand (n = 2), metatarsal (n = 1), fibula (n = 1), humerus (n = 2), and tibia (n = 1). The mean maximum tumor diameter was 29.7 mm (range: 11.8-64.0 mm), and the mean tumor volume was 9.8 mL (range: 0.4-40.7 mL). The amount of OCP/Gel used for filling after curettage ranged from 0.6 g to 16 g (mean: 5.9 g). Anhydrous ethanol treatment was applied in three cases, while two patients had a history of previous treatment. Three patients had episodes of preoperative pathological fracture (Table 1).

Postoperative radiographic follow-up demonstrated a gradual decrease in radiolucency within the grafted area over time. CT at six months confirmed the formation of trabecular bone structures at the graft site in all cases. One patient developed a suture abscess; however, no complications related to the bone graft substitute, such as infection, toxicity, or postoperative fracture, were observed. No apparent difference in early radiographic remodeling was observed between patients with pathological fractures and those without fractures.

**Figure 1:**
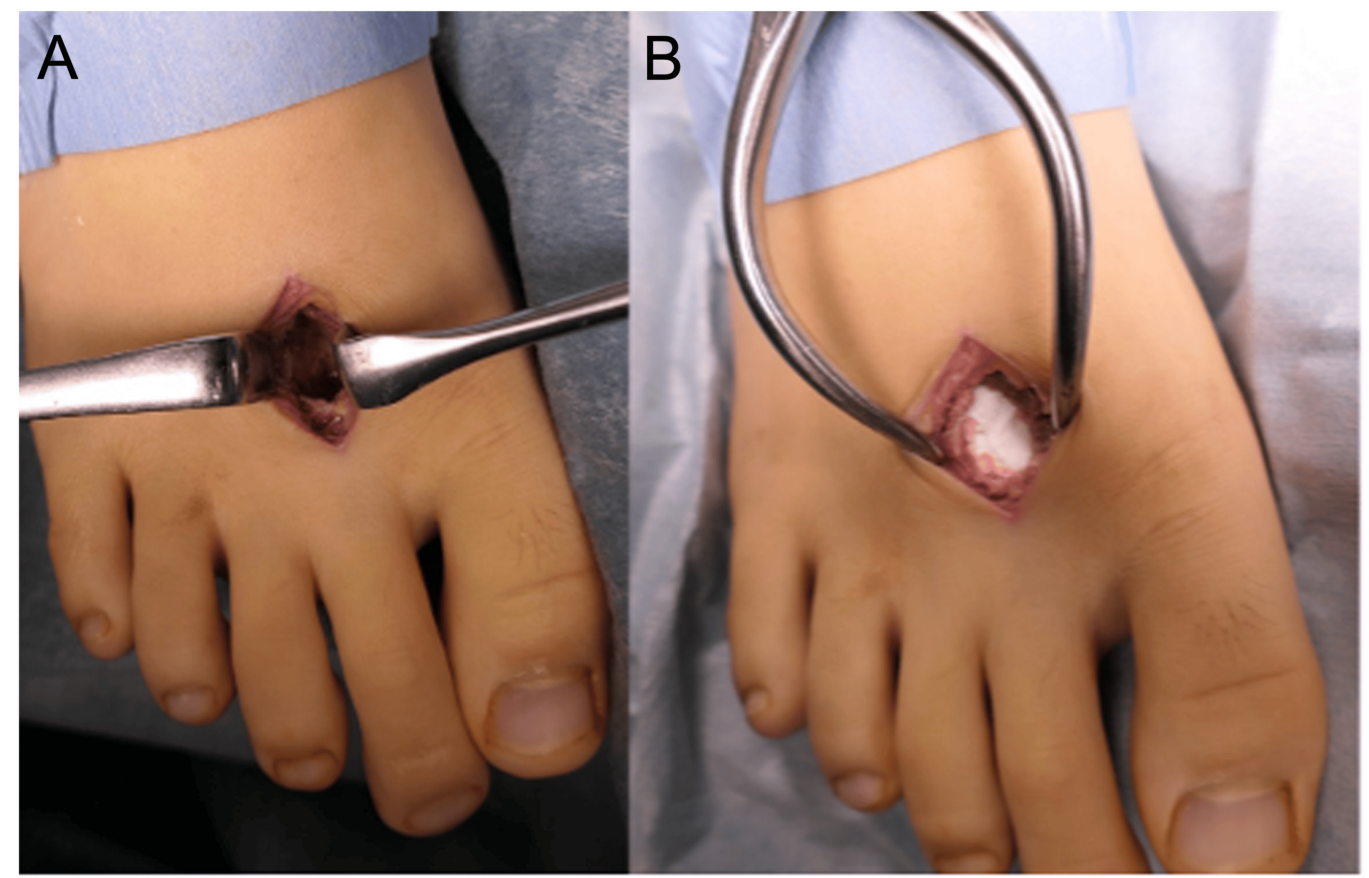
Intraoperative photographs Intraoperative photograph of a 12-year-old boy with a giant cell tumor of bone in the right second metatarsal. (A) After curettage. (B) After OCP/Gel grafting.

**Figure 2:**
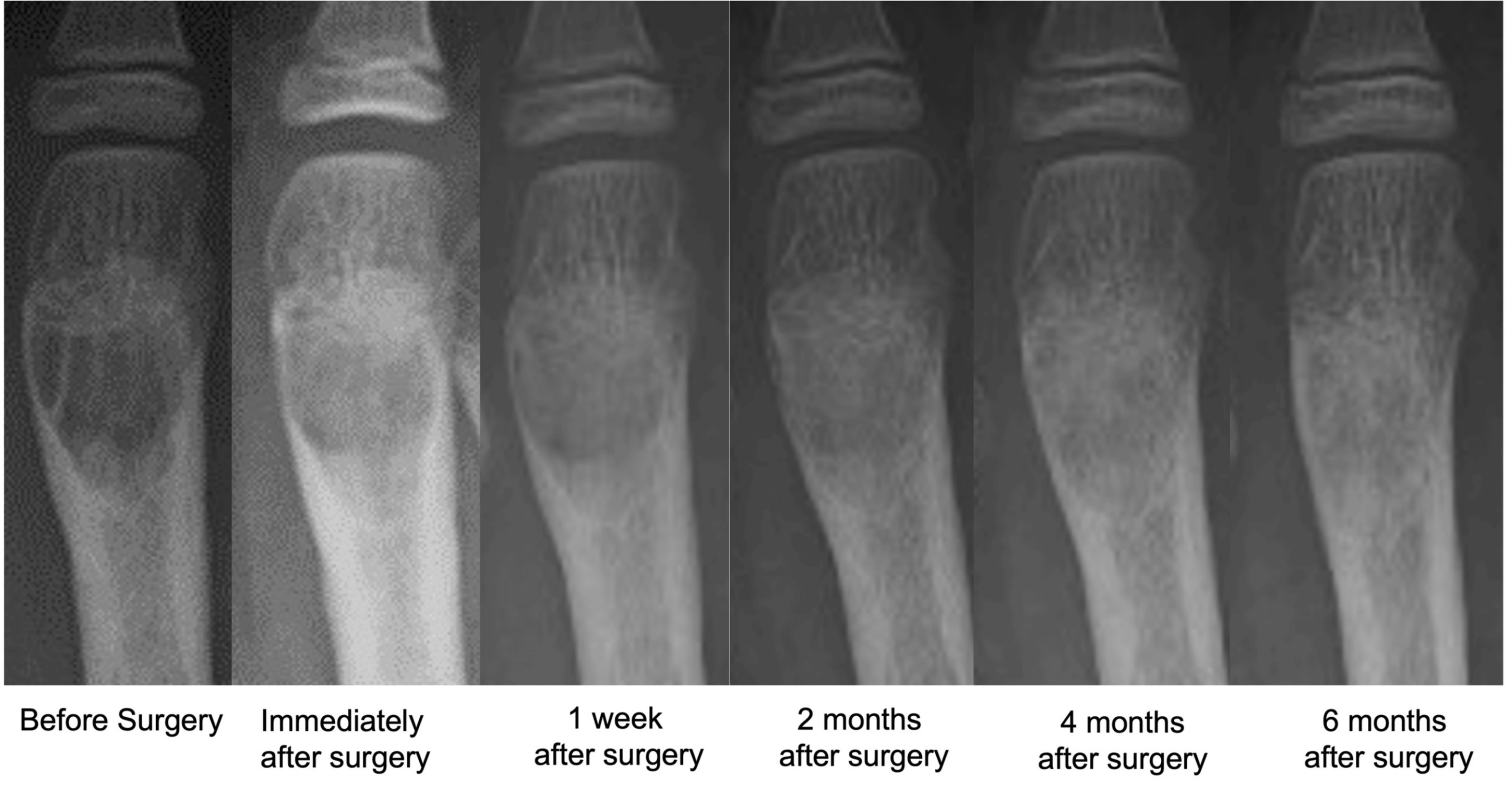
Serial radiographs of a patient with a giant cell tumor of bone Serial radiographs of a 12-year-old boy with a giant cell tumor of bone in the right second metatarsal.

**Figure 3:**
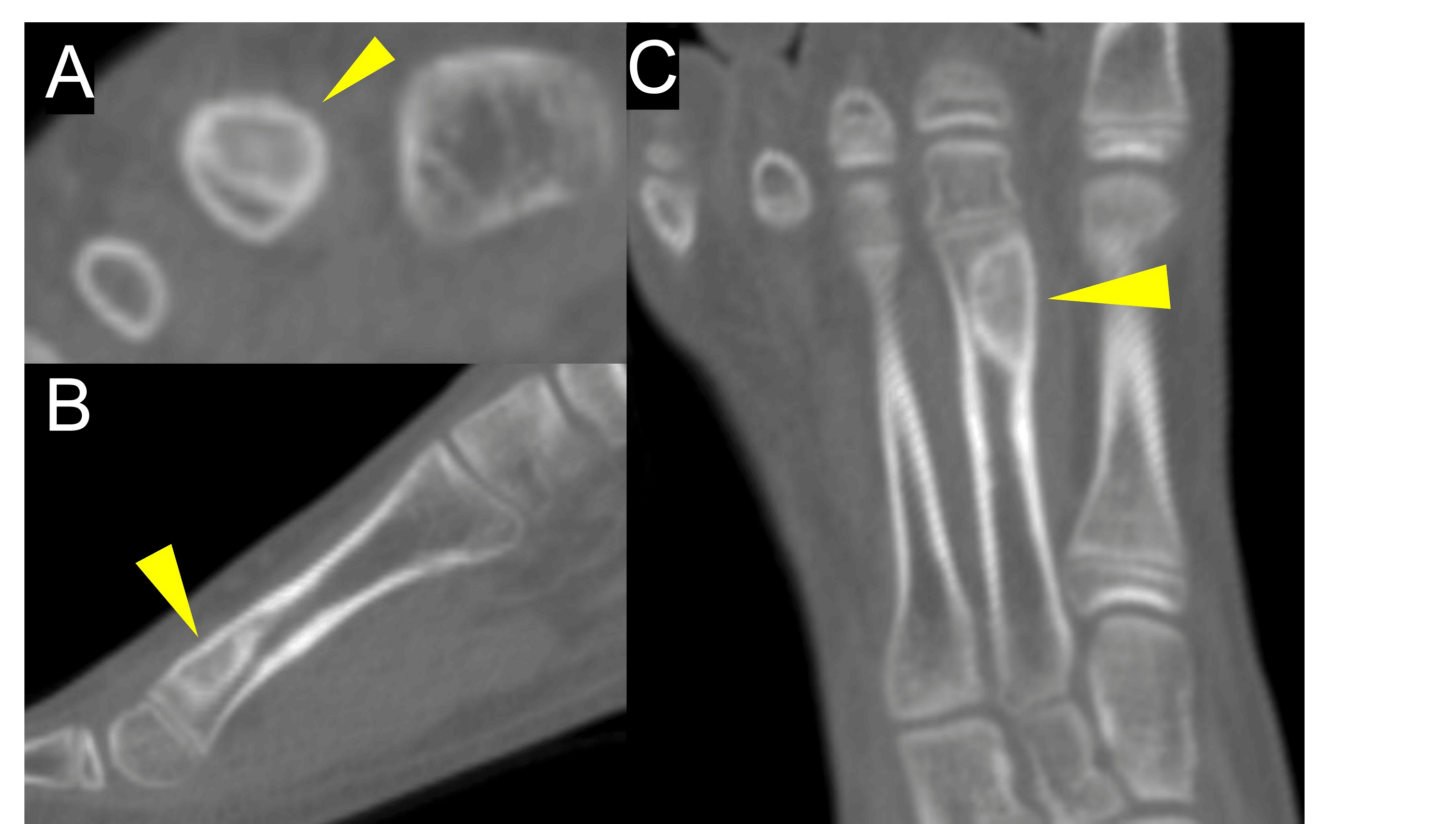
Computed tomography images obtained six months after surgery Computed tomography images obtained six months after surgery in a 12-year-old boy with a giant cell tumor of bone in the right second metatarsal. (A) Axial, (B) sagittal, and (C) coronal views. Arrowheads indicate the OCP/Gel graft site following tumor curettage.

A representative case involved a 12-year-old boy diagnosed with a giant cell tumor of bone at the distal end of the right second metatarsal. Following surgical curettage and grafting with the OCP/Gel (Figure 1), serial postoperative radiographs showed a progressive decrease in radiolucency at the graft site. At six months (Figure 2), evident trabecular bone formation indicated ongoing integration and remodeling. CT images obtained at the six-month follow-up (Figure 3) further confirmed the continuity of trabecular bone and new bone regeneration at the defect site.

## Discussion

Bone defect reconstruction after curettage for benign bone tumors requires bone substitutes that combine safety, availability, and a reliable capacity for bone regeneration. In this study, the use of OCP/Gel suggested favorable early bone formation without any material-related complications. Radiographic evaluation showed a gradual reduction in radiolucency followed by trabecular bone formation in all cases, suggesting that OCP/Gel was progressively resorbed and replaced by new bone within six months after implantation.

Autologous bone grafting remains the gold standard for bone reconstruction because of its superior osteoconductive and osteoinductive properties; however, donor site morbidity, limited graft volume, and prolonged operative time remain problematic [2,22,23]. Allogeneic grafts, although osteoconductive, carry the risk of infection and are rarely used in Japan due to cost and supply limitations [24,25]. Xenografts derived from bovine or porcine bone are primarily used in the dental field, but their application in orthopedics is limited by immunogenicity and concerns regarding zoonotic disease transmission [24]. These limitations have driven the development of synthetic bone substitutes that offer consistent quality, unlimited availability, and favorable biological performance.

Among synthetic materials, HA and β-TCP are widely used due to their biocompatibility and osteoconductivity; however, they lack osteoinductive potential and degrade slowly, which may hinder normal bone remodeling [2,4]. In contrast, OCP exhibits both osteoconductivity and intrinsic osteoinductivity [7,8,26], and its higher resorbability compared to HA and β-TCP facilitates dynamic replacement by newly formed bone [3,13]. OCP-based composites, such as OCP/Gel, combine these biological advantages with improved handling characteristics and have recently become clinically available in Japan.

The present clinical results align with previous preclinical studies that demonstrated the superior osteogenic performance of OCP/Gel in rat and rabbit cortical defect models, as well as in miniature swine spinal fusion [8,16,17]. The current findings suggest that OCP/Gel promotes rapid bone remodeling even in post-curettage cavities, where osteogenic potential may be compromised. Another notable advantage is the material’s low initial radiopacity, which enables clinicians to monitor the gradual replacement of the graft by new bone, unlike HA, which remains radiopaque for prolonged periods [4,24]. Importantly, no adverse reactions such as infection, foreign-body response, or postoperative fracture were observed, supporting the clinical safety of the material.

Nevertheless, OCP/Gel has limited initial mechanical strength compared with HA and β-TCP [24] and is therefore not suitable for immediate weight-bearing. It may be used in weight-bearing bones only when rigid fixation and postoperative weight-bearing restrictions are applied, rather than being inherently more suitable for such regions. In addition, variability in the ratio of graft weight to defect volume among cases likely reflects differences in packing density due to the sponge-like, compressible nature of OCP/Gel, as well as limitations of ellipsoid-based defect volume estimation for irregular cavities. Moreover, although limited by sample size, no clear differences in early radiographic remodeling were observed between patients with pathological fractures and those without.

This study has several limitations. First, the number of cases was small, and the follow-up period was relatively short, with only early radiographic outcomes assessed. Second, this was a single-center study, which may limit the generalizability of the findings. Third, the present cohort included relatively small bone defects; therefore, further evaluation in larger bone defects and load-bearing sites is warranted to determine the broader clinical applicability of OCP/Gel.

## Conclusions

This preliminary case series suggests that OCP/Gel promotes early radiographic bone formation and integration after curettage of benign bone tumors, with no major complications. Because only short-term radiographic outcomes were assessed, long-term structural and functional effects remain unknown. These findings support its potential as a safe and effective synthetic bone substitute for reconstructing bone defects after tumor surgery. Larger multicenter studies with long-term follow-up are needed to validate these preliminary results and to establish optimal indications for OCP/Gel in orthopedic practice.
